# Transcriptional events co-regulated by hypoxia and cold stresses in Zebrafish larvae

**DOI:** 10.1186/s12864-015-1560-y

**Published:** 2015-05-15

**Authors:** Yong Long, Junjun Yan, Guili Song, Xiaohui Li, Xixi Li, Qing Li, Zongbin Cui

**Affiliations:** The Key Laboratory of Aquatic Biodiversity and Conservation, Institute of Hydrobiology, Chinese Academy of Sciences, Wuhan, Hubei PR China; University of the Chinese Academy of Sciences, Beijing, PR China

**Keywords:** Zebrafish, Hypoxia, Cold stress, RNA-seq, Gene expression

## Abstract

**Background:**

Hypoxia and temperature stress are two major adverse environmental conditions often encountered by fishes. The interaction between hypoxia and temperature stresses has been well documented and oxygen is considered to be the limiting factor for the thermal tolerance of fish. Although both high and low temperature stresses can impair the cardiovascular function and the cross-resistance between hypoxia and heat stress has been found, it is not clear whether hypoxia acclimation can protect fish from cold injury.

**Results:**

Pre-acclimation of 96-hpf zebrafish larvae to mild hypoxia (5% O2) significantly improved their resistance to lethal hypoxia (2.5% O2) and increased the survival rate of zebrafish larvae after lethal cold (10°C) exposure. However, pre-acclimation of 96-hpf larvae to cold (18°C) decreased their tolerance to lethal hypoxia although their ability to endure lethal cold increased. RNA-seq analysis identified 132 up-regulated and 41 down-regulated genes upon mild hypoxia exposure. Gene ontology enrichment analyses revealed that genes up-regulated by hypoxia are primarily involved in oxygen transport, oxidation-reduction process, hemoglobin biosynthetic process, erythrocyte development and cellular iron ion homeostasis. Hypoxia-inhibited genes are enriched in inorganic anion transport, sodium ion transport, very long-chain fatty acid biosynthetic process and cytidine deamination. A comparison with the dataset of cold-regulated gene expression identified 23 genes co-induced by hypoxia and cold and these genes are mainly associated with oxidation-reduction process, oxygen transport, hemopoiesis, hemoglobin biosynthetic process and cellular iron ion homeostasis. The alleviation of lipid peroxidation damage by both cold- and hypoxia-acclimation upon lethal cold stress suggests the association of these genes with cold resistance. Furthermore, the alternative promoter of *hmbsb* gene specifically activated by hypoxia and cold was identified and confirmed.

**Conclusions:**

Acclimation responses to mild hypoxia and cold stress were found in zebrafish larvae and pre-acclimation to hypoxia significantly improved the tolerance of larvae to lethal cold stress. RNA-seq and bioinformatics analyses revealed the biological processes associated with hypoxia acclimation. Transcriptional events co-induced by hypoxia and cold may represent the molecular basis underlying the protection of hypoxia-acclimation against cold injury.

**Electronic supplementary material:**

The online version of this article (doi:10.1186/s12864-015-1560-y) contains supplementary material, which is available to authorized users.

## Background

The concentration of dissolved oxygen (DO) and temperature are the most important environmental variables that affect the overall biological processes of fishes. Temperature limits the rates of cellular biochemical reactions and dictates all aspects of fish life, including metabolism, development, growth, reproduction and behavior [1]. Adverse effects and even death can be caused when water temperature falls outside the species-specific thermal tolerance range [1,2]. Molecular oxygen is used by all eukaryotic cells as the terminal electron acceptor in aerobic energy production and the presence of adequate oxygen is essential to the survival of nearly all vertebrates [3]. Oxygen deficiency can impair cellular energy generation, induce the formation of reactive oxygen species (ROS) and lead to cell damage and apoptosis [4-7]. In water environments, hypoxia (DO < 2 mg/L) often occurs due to the inherent properties of water and the rapid fluctuations in the pattern of oxygen production and consumption [8,9]. Exposure of fish to hypoxia can suppress development, reduce growth, disturb endocrine function, impair reproductive performance, and cause mass mortality for wild populations [9,10].

Due to the importance of oxygen and temperature to their life, fishes have evolved versatile mechanisms to acclimate oxygen deficiency and temperature variations in their habitats [11]. Pre-acclimation of fishes to moderate hypoxia or thermal stress can activate the acclimation pathways and increase the tolerance to lethal hypoxia or thermal stress [12-14], respectively. The rationale of hypoxia acclimation in fish is to increase oxygen uptake and reduce oxygen demands. Upon hypoxia, fishes usually skim the surface water containing more oxygen, increase ventilation volume to absorb more oxygen, or reduce motility to spare oxygen consumption [15,16]. Except for behavioral actions, extensive physiological and biochemical modifications such as gill modifications to increase surface area [17], increases in heart rate and hemoglobin content [18,19], alterations in the structure or activity of specific ion channels [20], and activation of anaerobic ATP production via glycolysis [21], are also involved in the process of hypoxia acclimation. The acclimation of fish to thermal stresses was considered to be a process of “biochemical restructuration” [22], including synthesizing temperature specific isoenzymes [22], increasing the content of membrane lipid and the degree of fatty acid unsaturation [23], recruiting different muscle fiber types [24], generating molecular chaperones [25], and changing mitochondrial densities and their properties [26].

Numerous studies have focused on the molecular mechanisms underlying the acclimation responses to environmental stressors including hypoxia and temperature fluctuations in the last decade. Hypoxia-regulated gene expression in the embryos and adult tissues of zebrafish (*Danio rerio*) [27-30], goby (*Gillichthys mirabilis*) [31], medaka (*Oryzias latipes*) [32] and *Xiphophorus maculatus* has been characterized with microarrays [33]. Transcriptional responses to thermal stresses in species such as zebrafish [34], common carp (*Cyprinus carpio*) [35], channel catfish (*Ictalurus punctatus*) [36], annual killifish (*Austrofundulus limnaeus*) [37], coral reef fish (*Pomacentrus moluccensis*) [38], rainbow trout (*Oncorhynchus mykiss*) [39] and Antarctic plunderfish (*Harpagifer antarcticus*) [40] have been investigated using microarray as well. Very recently, RNA-seq was applied to explore the transcriptional responses of fish to hypoxia and thermal stress at the whole genome level [12,41,42]. These studies have demonstrated that hypoxia and thermal stresses can induce profound changes in gene expression profiles. Although the master factors responsible for mediating temperature stress-regulated gene expression remain unknown, hypoxia-inducible factor-1 (HIF-1) was revealed to be the key regulator for hypoxia-induced genes [43].

Effects of hypoxia and thermal stresses on fishes are often examined separately, but an increasing body of evidence indicates the interaction between temperature and oxygen on organismal performance [44]. Exposure of fish to both low and high temperature stresses can impair cardiovascular functions and decrease circulatory oxygen concentration, thus induce tissue hypoxia in spite of ample oxygen supply from the environment [45]. Therefore, oxygen was considered to be the limiting factor for thermal tolerance of fish due to restrictions in cardiovascular performance at extreme temperatures [45-47]. Thermal limitation in fish was interpreted as being caused first by limited oxygen supply capacity and second by transition to anaerobic metabolism [48]. Accordingly, acclimation of channel catfish to hypoxia can enhance the tolerance to acute heat stress through improving cardiovascular performance [19,49]. Furthermore, exposure of zoarcid fish (*Zoarces viviparus*) [50] and crucian carp (*Carassius carassius*) [51] to cold stress induced the expression and DNA binding activity of HIF-1, suggesting the activation of hypoxia-induced pathways by cold stress. However, it remains unclear whether fish can develop a cross-resistance to hypoxia and cold and how multiple-stress responses are co-regulated.

Zebrafish is a good model to study the mechanisms of environmental acclimation and acclimation to both hypoxia and cold has been previously reported in this species [12,13]. Recently, RNA-seq gradually substituted the microarray approach in exploring transcriptional responses of organisms to environmental stressors due to its high sensitivity and accuracy, digital expression and the ability to distinguish transcript isoforms [52]. Except for detecting transcript abundance, RNA-seq identifies alternative splicing and alternative promoter usage events at the same time and provides a more holistic view of the transcriptome [52]. Although hypoxia-regulated gene expression has been extensively investigated in zebrafish, it remains to be characterized by RNA-seq. In this study, we investigated the effect of hypoxia acclimation on cold tolerance and *vice versa* in zebrafish larvae, characterized the transcriptional responses to hypoxia at the whole-genome level using RNA-seq, and made a comparison of hypoxia- and cold-induced transcriptomes.

## Results

### Hypoxia acclimation increased the cold resistance of zebrafish larvae

**Figure 1:**
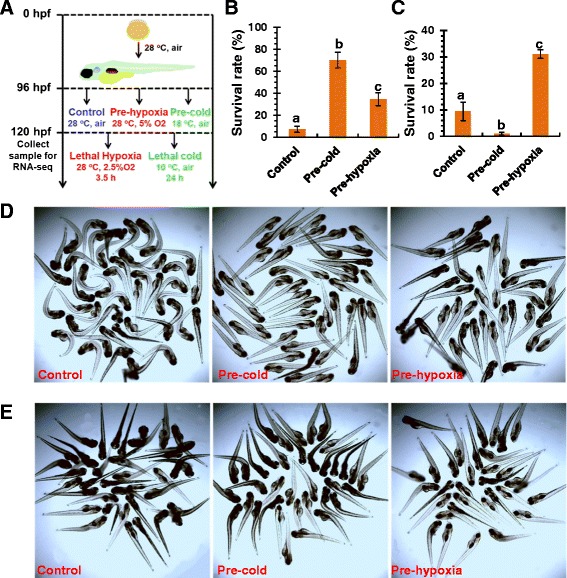
Pre-acclimation to hypoxia increased the cold resistance of zebrafish larvae. **(A)** Flowchart of stressor exposure. Zebrafish embryos were incubated in air at 28°C from fertilization to 96 hpf and then pre-acclimated to hypoxia or cold for 24 h. Sample collection for RNA-seq and lethal hypoxia and cold exposure were performed at 120 hpf. **(B and C)** Survival rates of zebrafish larvae after lethal cold **(B)** and hypoxia **(C)**. Data was shown as mean ± standard deviation (n = 5). Different letters above the error bars indicate significant difference (p < 0.05) between treatment groups. **(D and E)** Photographs of zebrafish larvae after lethal cold **(D)** and hypoxia **(E)** challenge. Dead fish displayed an obvious body curvature.

To investigate the cross-resistance between hypoxia and cold stress, zebrafish larvae pre-acclimated to mild hypoxia or cold were exposed to lethal hypoxia and cold, respectively (Figure 1A). Zebrafish larvae exposed to 5% O2 for 24 h demonstrated smaller intestine lumen, larger yolk sac and smaller body length when compared to the control larvae maintained in air (Additional file 1), demonstrating an obvious effect of hypoxia exposure. As shown in Figure 1B and C, both hypoxia and cold acclimation enhanced the resistance of zebrafish larvae to the lethal level of the same stressor, indicating the activation of protective mechanisms upon mild hypoxia and cold. Moreover, hypoxia acclimation significantly increased the survival rate of zebrafish larvae after lethal cold exposure (Figure 1B and D), suggesting hypoxia-inducible signaling pathways in zebrafish larvae are involved in the development of cold resistance. However, pre-exposure to cold significantly decreased the survival rate of larvae upon lethal hypoxia exposure (Figure 1C and E) and no mortality was observed when the pre-cold acclimated larvae were suddenly transferred to 28°C incubation in an additional experiment (data not shown). Therefore, instead of enhancing resistance, cold-acclimation sensitized the larvae to hypoxia stress.

### Hypoxia-regulated gene expression

In order to disclose the molecular basis underlying the protective effect of hypoxia acclimation on zebrafish larvae against lethal cold stress, gene expression in larvae acclimated to 5% O2 for 24 h was characterized with RNA-seq. The low quality reads were filtered and the remaining clean reads were then mapped to the zebrafish genomic sequence. The statistics for read pre-processing and mapping were displayed in Table 1. The total number of raw read pairs ranged from 19.80 to 30.53 million and about 96% of the raw reads passed the quality threshold. About 91% of the clean reads were mapped to the genomic sequence by Tophat and the number of reads mapped to splice junctions was quite similar among different samples (from 5.22 to 5.26 million). Finally, 75.51 - 77.95% of the alignment was unique in the genome. These results suggest the high quality of our sequencing datasets.

**Table 1:** **Statistics for read filtering and mapping**

**Sample name**	**Control1**	**Control2**	**Control3**	**Hypoxia**	**Hypoxia**	**Hypoxia**
Total reads (M)	19.80x2	24.72x2	19.48x2	30.53x2	26.85x2	28.45x2
Good reads (M)	38.05	47.76	37.62	58.76	51.94	54.91
% Good reads	96.09	96.61	96.58	96.24	96.72	96.52
Processed reads (M)	37.52	47.22	37.18	57.94	51.36	54.44
Mapped reads (M)	34.46	42.89	33.99	53.08	47.28	50.00
% Mapped (M)	91.84	90.83	91.42	91.61	92.06	91.84
Total alignment (M)	43.68	53.52	43.79	68.4	58.95	62.26
Total potential splices (M)	5.22	5.25	5.22	5.26	5.24	5.24
% Reads mapped to junction	15.15	12.24	15.36	9.91	11.08	10.48
Unique mapping (M)	33.35	41.72	33.54	51.65	45.49	48.24
% Unique mapping	76.35	77.95	76.59	75.51	77.17	77.48

**Figure 2:**
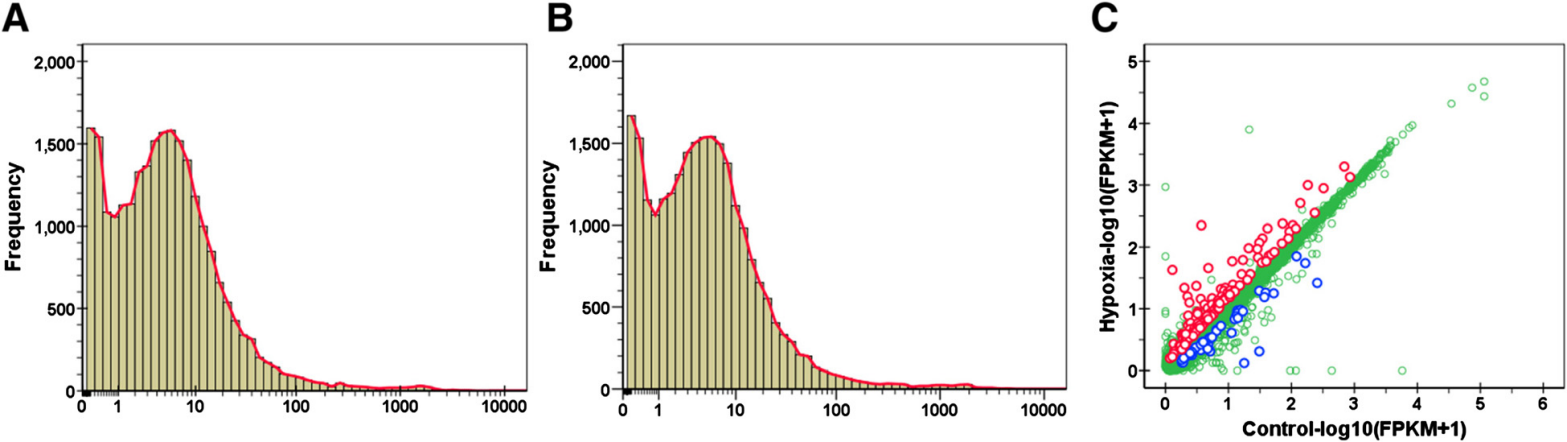
Gene expression regulated by hypoxia. **(A and B)** Distribution of FPKM values for genes expressed in the control **(A)** and hypoxia-acclimated **(B)** zebrafish larvae. The red interpolation line denotes a bimodal distribution of the frequency of FPKM. **(C)** Correlation of gene expression between the control and hypoxia-treated group. The up- and down-regulated genes were shown in red and blue, respectively. Genes not regulated by hypoxia treatment were shown in green.

After read mapping, gene expression was calculated using Cufflinks and the abundances of genes were expressed as FPKM (Fragments per kilobase of transcript per million fragments mapped) [53]. As shown in Figure 2A and B, most of the genes were of low and medium abundance according to their FPKM values and no obvious difference was found in the abundance distribution curve between the control and hypoxia-acclimated samples, suggesting a smaller effect of hypoxia on gene expression when compared to cold stress [12]. The correlation between the abundance of genes under control and hypoxia is displayed in Figure 2C. Genes with a fold change ≥ 1.5 and a q-value ≤ 0.05 were considered to be differentially expressed. The number of up- and down-regulated genes was 132 and 41, respectively (Additional file 2).

The most prominent hypoxia-induced gene was *hbz* (hemoglobin zeta), followed by *ponzr4* (plac8 onzin related protein 4), *egln3* (egl nine homolog 3), *hpx* (hemopexin), *ponzr3* (plac8 onzin related protein 3), *hbm* (hemoglobin, mu), *alas2* (aminolevulinate, delta-, synthetase 2), *hbbe2* (hemoglobin beta embryonic-2), *ankrd37* (ankyrin repeat domain 37), *p4ha1b* (prolyl 4-hydroxylase, alpha polypeptide I b) and two uncharacterized genes *si:dkey-202 l22.6* and *loc100537766*. These genes were up-regulated more than 5-fold by hypoxia (Additional file 2). The gene most highly inhibited by hypoxia was *he1a* (hatching enzyme 1a), the expression of which was reduced 51-fold by hypoxia. The expression of genes including *lifrb* (leukemia inhibitory factor receptor alpha b), *clca1* (chloride channel accessory 1) *loc100537819* and *si:ch73-362 m14.3* was inhibited 3-fold upon hypoxia (Additional file 2).

### Validation of RNA-seq data with qPCR

The qPCR assays were performed to validate the RNA-seq results. To identify internal reference genes appropriate for hypoxia exposure, two commonly used reference genes (*actb1* and *rpl13a*) and seven genes (*ada*, *smarce1*, *erp44*, *ube2e1*, *gnb1b*, *rbx1* and *yipf3*) demonstrating considerable expression stability after hypoxia treatment were selected as candidates (Additional file 3). The relative expression of these genes was detected by qPCR and their expression stability upon hypoxia exposure was analyzed using geNorm [54] and Normfinder [55], respectively. Both geNorm and Normfinder revealed that *smarce1*and *erp44* are the most stable genes (Additional file 4). Therefore, the geometric average of their expression was used as normalization factor for the analysis of hypoxia-related qPCR data.

**Figure 3:**
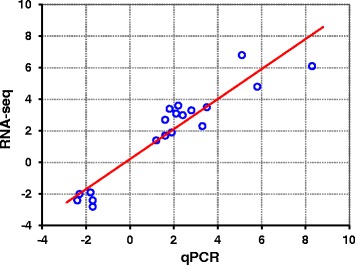
qPCR Validation of RNA-seq data. Expression of genes detected by RNA-seq was plotted against that of qPCR. The reference line indicates the linear correlation between the results of RNA-seq and qPCR.

The expression of 18 genes (two transcripts of *hmbsb* were analyzed separately) was selected to be detected by qPCR to validate the RNA-seq data. As shown in Table 2 and Figure 3, results from qPCR agreed very well with those of RNA-seq for both up- and down-regulated genes. A Spearman bivariate correlation analysis revealed that the data of RNA-seq and qPCR was significantly correlated (p < 0.01, correlation coefficient = 0.888), indicating the reliability of RNA-seq data.

**Table 2:** **Validation of RNA-seq data with qPCR**

**Gene symbol**	**Fold change**	
	**RNA-seq**	**qPCR**
*hmbsb-*P1	1.2	1.4
*hmbsb-*P2	5.8	4.8
*ak3*	1.6	2.7
*cpox*	2.8	3.3
*hif1an*	2.2	3.6
*ncoa4*	1.6	1.7
*osgn1*	1.8	3.4
*hbm*	8.3	6.1
*hephl1*	1.9	1.9
*mb*	3.3	2.3
*steap3*	3.5	3.5
*p4ha1b*	5.1	6.8
*tfr1a*	2.1	2.6
*urod*	2.4	3.0
*fads2*	−2.3	−2.0
*mep1b*	−2.4	−2.4
*elov2*	−1.8	−1.9
*slc34a2a*	−1.7	−2.8
*slc13a2*	−1.7	−2.4

### Functional classification of hypoxia-regulated genes

**Figure 4:**
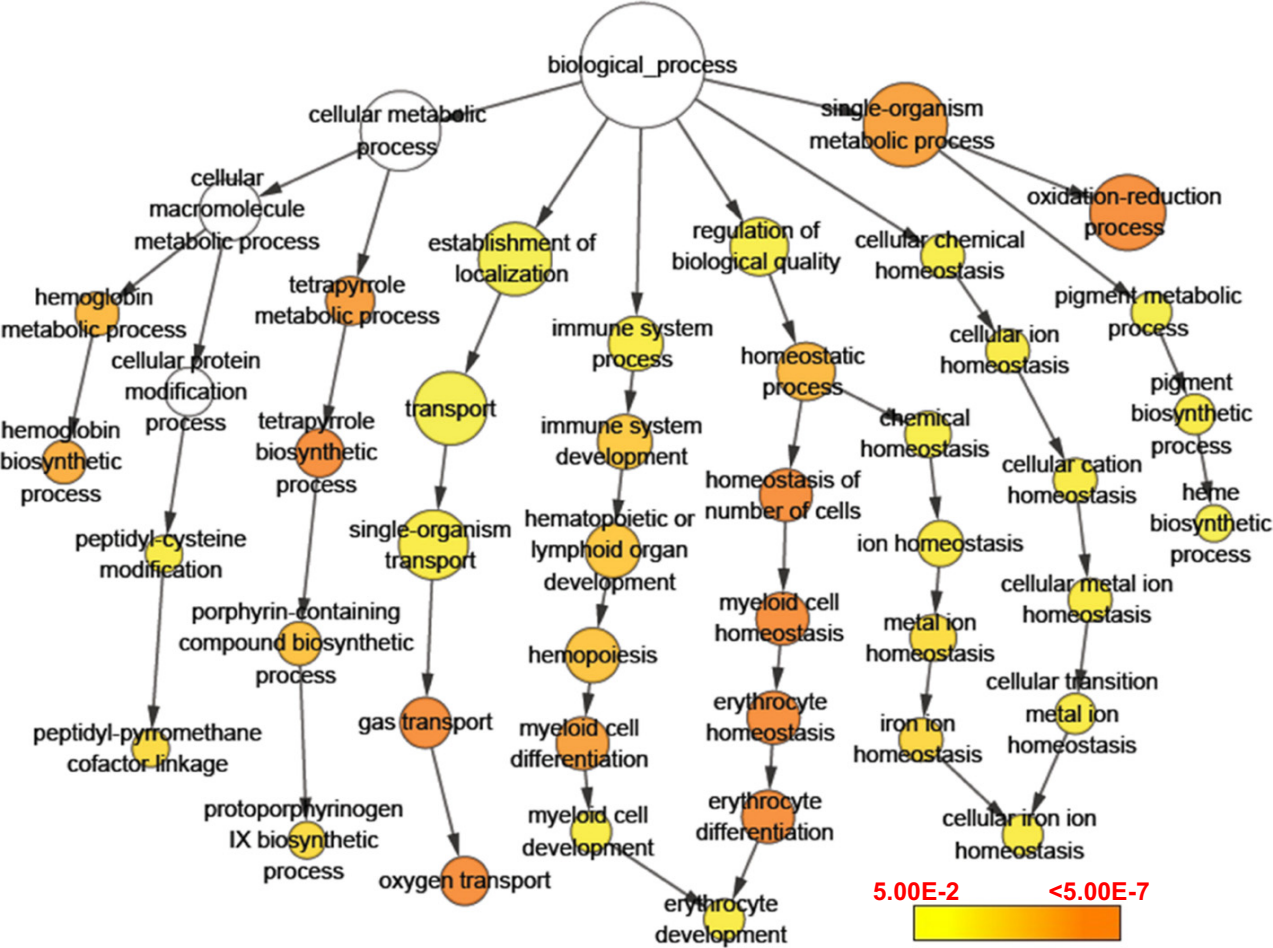
GO enrichment analysis of genes up-regulated by hypoxia. The size of circles is proportional to the number of genes associated with the GO term. The arrows represent the relationship between parent–child terms. The color scale indicates corrected p-value of enrichment analysis.

GO enrichment analyses indicated that the most enriched biological processes for hypoxia-induced genes include oxygen transport, oxidation-reduction process, hemoglobin biosynthetic process, erythrocyte development, cellular iron ion homeostasis, protoporphyrinogen IX biosynthetic process and peptidyl-pyrromethane cofactor linkage (Figure 4 and Additional file 5). These enriched processes are tightly associated with oxygen uptake and delivery, which is consistent with the demand to enhance oxygen uptake and delivery efficiency upon hypoxia. Hypoxia-inhibited genes are mainly involved in inorganic anion transport, sodium ion transport, very long-chain fatty acid biosynthetic process, glycolysis and cytidine deamination (Additional file 6).

### Genes co-regulated by hypoxia and cold stress

**Figure 5:**
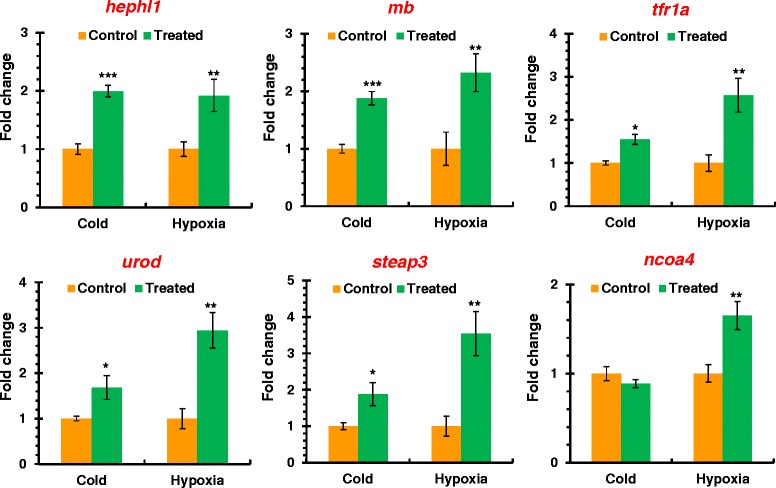
Genes co-induced by hypoxia and cold. The expression of genes in zebrafish larvae after hypoxia and cold pre-acclimation was characterized using qPCR. Data was shown as mean ± standard deviation (n = 3). Significant differences between control and hypoxia or cold exposed samples were demonstrated using asterisks. “*” p < 0.05, “**” p < 0.01 and “***” p < 0.001.

The protection of hypoxia-acclimation against adverse effects elicited by lethal cold prompts us to characterize the transcriptional events co-regulated by hypoxia and cold. A comparison with our previous cold-regulated gene expression dataset [12] identified 23 genes that were induced by both hypoxia and cold (Table 3). The expression of six genes induced by both hypoxia and cold exposure were investigated using qPCR to validate the co-regulation. As shown in Figure 5, the expression of *hephl1* (hephaestin-like 1), *mb* (myoglobin), *tfr1a* (transferrin receptor 1a), *urod* (uroporphyrinogen decarboxylase) and *steap3* (STEAP family member 3, metalloreductase) was up-regulated upon both hypoxia and cold. A search for the function annotation in the gene ontology database (http://geneontology.org/) indicates that these co-regulated genes are mainly involved in oxidation-reduction process, oxygen transport, hemopoiesis, hemoglobin biosynthetic process and cellular iron ion homeostasis (Table 3), suggesting that the activation of these processes are associated with the hypoxia-induced protection against cold stress.

**Table 3:** **Genes co-induced by hypoxia and cold**

**Genes**	**Fold change**		**Biological process**
	**Hypoxia**	**Cold**	
*cyp46a1*	2.54	5.30	Oxidation-reduction process
*hephl1*	1.88	2.52	Oxidation-reduction process
*sqrdl*	1.66	2.19	Oxidation-reduction process
*bbox1*	1.60	1.64	Oxidation-reduction process, carnitine biosynthetic process
*cox7a2*	1.60	1.63	Oxidation-reduction process, proton transport, ion transmembrane transport
*cox6b1*	1.90	1.51	Oxidation-reduction process, proton transport, ion transmembrane transport
*mb*	3.32	1.98	Oxygen transport,vasculogenesis
*hbae3*	2.88	1.62	Oxygen transport
*tspo*	1.53	1.58	Hemopoiesis, primitive erythrocyte differentiation
*tfr1a*	2.19	1.63	Hemoglobin biosynthetic process, hemopoiesis, erythrocyte differentiation
*tfr1b*	1.76	2.28	Hemoglobin biosynthetic process, cellular iron ion homeostasis, proteolysis
*hmbsa*	1.94	1.96	Heme biosynthesis, tetrapyrrole biosynthetic process
*urod*	2.46	1.75	Heme biosynthetic process, protoporphyrinogen IX biosynthetic process
*steap3*	3.49	2.19	Transferrin transport, cellular iron ion homeostasis, protein secretion
*slc16a1*	1.61	1.85	Monocarboxylic acid transport, organic anion transport
*abcb10*	1.61	1.96	Transmembrane transport, ATP catabolic process
*acta1b*	1.59	1.92	Embryonic heart tube development
*klf4b*	1.57	1.53	Hatching gland development, erythrocyte differentiation, hemopoiesis
*ncoa4*	1.64	1.61	Positive regulation of transcription, DNA-dependent
*eno1a*	1.51	1.59	Glycolysis/Gluconeogenesis
*zgc:161979*	1.71	6.10	No data
*zgc:113232*	1.79	2.83	No data
*znf292b*	1.50	1.77	No data

### Alternative promoter usage of *hmbsb* upon hypoxia and cold

**Figure 6:**
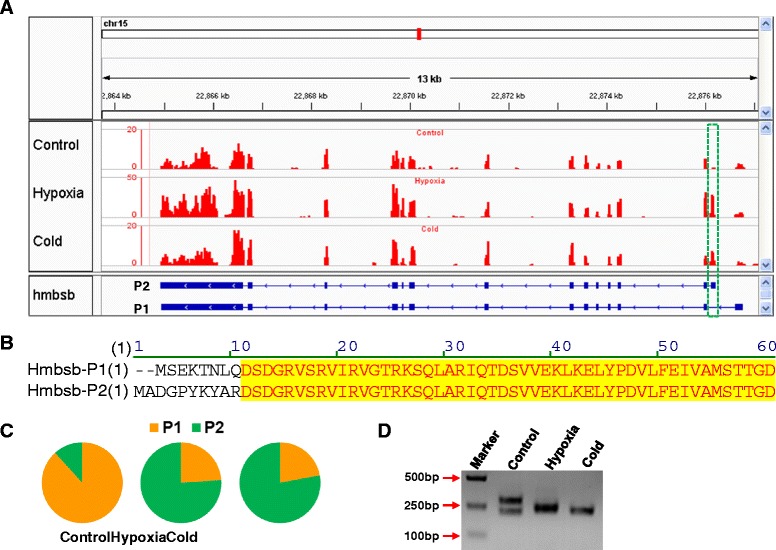
Alternative promoter usage of *hmbsb* upon hypoxia and cold. **(A)** Read coverage at *hmbsb* locus. The upper panel shows the read coverage of representative sample from each treatment group and the bottom panel indicates the structure of *hmbsb* transcripts. The green box indicates the first exon determined by the alternative promoter. **(B)** Partial sequence alignment of Hmbsb peptides. **(C)** Relative abundance of *hmbsb* transcripts detected by RNA-seq. **(D)** 5′ ends of *hmbsb* cDNAs.

Except for differential gene expression, alternative promoter usage of *hmbsb* (hydroxymethylbilane synthase b) upon hypoxia was identified by Cufflinks. Dataset comparison demonstrated that promoter transition of *hmbsb* was also induced by cold stress. Due to the significance of alternative promoter usage in transcriptome diversity, we analyzed this event in detail and confirmed it using 5′ RACE. Hmbsb is involved in the biosynthetic process of tetrapyrrole, a source material of heme biosynthesis. Alternative promoter usage of *hmbsb* results in alternative first exons that are spliced to the common second exon and the start codon is present within the alternative first exon (Figure 6A). The corresponding transcripts of alternative promoters (P1 and P2) were designated as hmbsb-P1 and hmbsb-P2, respectively. The read coverage of the first exon for hmbsb-P2 was obviously higher in hypoxia and cold-treated samples than that of control (Figure 6A). The alternative promoter usage of *hmbsb* results in different N-terminus of encoded peptides (Figure 6B). Hmbsb-P1 is the main transcript under control condition; however, the main transcript was changed to hmbsb-P2 when exposed to hypoxia and cold (Figure 6C).

**Figure 7:**
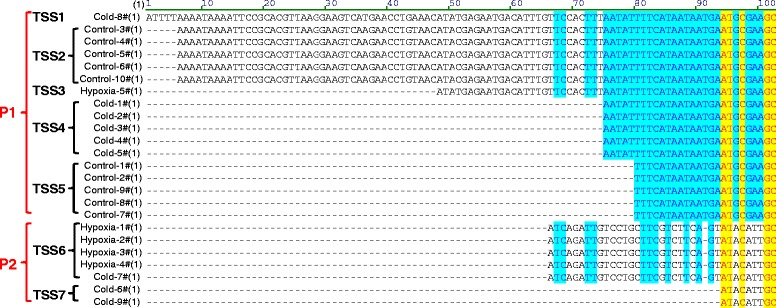
Transcription start sites of *hmbsb* under different conditions. Total RNA samples from different treatment group were subjected to 5′ RACE, respectively. The 5′ ends of *hmbsb* cDNA were cloned and sequenced. The obtained sequences were aligned and classified into promoter and transcription start site groups.

The 5′RACE assay based on RNA adaptor ligation was performed to analyze the 5′ ends of *hmbsb* cDNAs. Electrophoresis identified two bands for the control and one band for both hypoxia- and cold-treated samples (Figure 6D). The resulted PCR fragments of different samples were cloned and sequenced. A total of seven transcription start sites (TSSs) were identified for the two alternative promoters. TSS1 to TSS5 is determined by P1 and TSS6 and TSS7 belong to P2 (Figure 7). Consistent with Figure 6D, all clones from the control sample represented transcripts driven by P1 (initiated from TSS2 or TSS5) and the transcript of TSS2 is 74 base pairs longer than that of TSS5. P2 was hypoxia- and cold-specific and most of the clones from the hypoxia-treated sample were transcripts driven by P2 (4/5) and the ratio of P1/P2 for clones derived from the cold-treated sample was 3/6 (Figure 7). These results indicate that the alternative promoter P2 of *hmbsb* gene is specifically activated by hypoxia and cold and the activating effect of hypoxia is stronger than that of cold.

### Cold and hypoxia pre-acclimation alleviated lipid peroxidation damage

**Figure 8:**
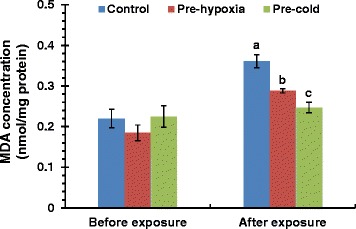
Cold- and hypoxia-acclimation alleviated oxidation damage upon lethal cold exposure. Larvae at 96 hpf were subjected to cold- or hypoxia-acclimation for 24 h and then incubated at 10°C for 12 h. The larvae in the same plate were homogenized for lipid peroxidation assays with a kit based on the reaction of thiobarbituric acid (TBA) with malondialdehyde (MDA). The MDA content was normalized to protein concentration. Data was shown as mean ± standard deviation (n = 3). Different letters above the error bars indicate significant differences (p < 0.05) among treatment groups.

Since most of the cold and hypoxia co-regulated genes are involved in improving cellular oxygen availability and oxidation-reduction process, we performed lipid peroxidation assays to test whether cold or hypoxia-acclimation can alleviate oxidation damage upon lethal cold exposure. The results indicate that exposure to mild cold or low oxygen stress has no effect on the content of malondialdehyde (MDA), the product of lipid peroxidation (Figure 8). After the exposure of larvae to lethal cold for 12 h, the MDA concentration of cold- and hypoxia-acclimated samples was significantly lower than that of the control (Figure 8). This is consistent with the protective effect of cold- and hypoxia-acclimation against lethal cold stress.

## Discussion

Oxygen and temperature are the most important environmental factors for the life of fishes and both hypoxia and temperature stress can cause deleterious effects on the organismal performance. Due to the importance of these factors, fishes have developed acclimation mechanisms to survive the daily and seasonal fluctuations in oxygen concentration or temperature in their habitats during the process of evolution. The interdependence between oxygen availability and thermal resistance has been well documented in fishes, i.e. exposure of fish to both low and high temperature stresses can induce tissue hypoxia through impairing the cardiovascular function and oxygen will subsequently limit the thermal tolerance of fish [45-47]. Although the protection of hypoxia acclimation against the adverse effect of heat stress has been reported [19,49], it is not clear whether there is a cross-resistance between hypoxia and cold stress. In this study, pre-acclimation of zebrafish larvae to mild hypoxia significantly increased the survival rates after lethal cold exposure, indicating the roles of hypoxia-inducible pathways in the establishment of cold resistance in fish. However, pre-acclimation to mild cold significantly reduced the survival rate of zebrafish larvae upon lethal hypoxia exposure. This is consistent with a previous study in mammal that cold acclimation decreased the hypoxia resistance of rats [56]. The increased incidence of fatty changes in the striated muscle and the marked depletion of liver glycogen of cold-acclimated rats upon low oxygen may be responsible for the hypoxia-susceptibility [56].

To investigate the molecular mechanisms underlying the protection of hypoxia-acclimation against cold stress, gene expression in hypoxia-acclimated zebrafish larvae was characterized using RNA-seq and compared with that of cold-acclimated samples. Although hypoxia-elicited gene expression in zebrafish has been explored by microarray in several previous studies [27-30], RNA-seq characterization may give new information due to its advantages over microarray. A total of 173 genes were found to be regulated by hypoxia in 96-hpf zebrafish larvae, including 132 up-regulated and 41 down-regulated genes. A large part of these genes were not found to be regulated by hypoxia in previous microarray studies. The number of genes influenced by hypoxia in this study is markedly smaller than that of cold-regulated genes [12], but is quite similar with the previous studies characterizing hypoxia-regulated gene expression using microarray [27,28]. This is consistent with the partial protection of hypoxia-acclimation against cold stress. It is possible that more complicated mechanisms are involved in the development of cold tolerance in fish.

Most of the hypoxia-induced genes identified in this study are involved in biosynthesis of the source material of hemoglobin, hematopoiesis and oxidation-reduction processes. Hemoglobin genes including *hbz*, *hbm*, *hbae1* (hemoglobin alpha embryonic-1), *hbae3* (hemoglobin alpha embryonic-3), *hbbe1.1* and *hbbe2* were among the most prominent gene families induced by hypoxia (Additional file 2). Although *hbz* was the most highly up-regulated gene under hypoxia, *hbae3*, *hbbe2* and *hbbe1.1* were the most abundant hemoglobin genes in both control and hypoxia-treated samples. However, hemoglobin genes were not found to be up-regulated in 24-hpf zebrafish embryos exposed to hypoxia [27]. Genes involved in erythrocyte differentiation such as *alas2* (aminolevulinate, delta-, synthetase 2), *tfr1a* (transferrin receptor 1a), *slc4a1a* (solute carrier family 4, anion exchanger, member 1a) and *gata1a* (GATA binding protein 1a) were also highly induced by hypoxia. *ALAS2* encodes 5-aminolevulinate synthase, the rate-controlling enzyme of erythroid heme synthesis; mutations of this gene are the causative of the X-linked sideroblastic anemia in human [57]. Transferrin receptor TRF1 plays a crucial role in cellular iron uptake and is previously reported to be induced by hypoxia in human cell lines [58]. Anion exchanger SLC4A1 functions as a transporter that mediates anion exchange across the cell membrane and as a structural protein, which is required for normal flexibility and stability of the erythrocyte membrane via the interactions of its cytoplasmic domain with cytoskeletal proteins [59]. Hematopoietic transcription factor GATA1 is indispensible for the maturation of erythrocytes [60]. The up-regulation of these genes underlies the increased resistance of zebrafish larvae to lethal hypoxia during the acclimation process.

HIF-1 is the critical regulator for hypoxia response and plays key roles in cellular hypoxia acclimation [61]. The activity of HIF-1 is tightly regulated by the *egl-9* family hypoxia-inducible factors (Egln) and the hypoxia-inducible factor 1 alpha subunit inhibitor (Hif1an). Egln genes encode proline hydroxylases (PHD) which mediate proteosomal degradation of HIF1α under normoxic oxygen conditions [62]. Egln genes were reported to be regulated by HIF-1 and suggested to be a negative feedback regulatory mechanism for limiting accumulation of HIF1α in hypoxia [63]. Like Egln members, Hif1an (also known as factor inhibiting HIF, FIH) hydroxylates a conserved asparaginyl residue within HIF1α when oxygen is available, and thus prevents the recruitment of co-activators and suppresses the activity [64]. Because the activities of both Egln members and Hif1an are oxygen-dependent, HIF1α is stabilized under hypoxia and dimerizes with HIF1β to form active HIF-1. Zebrafish has four *hif1a* genes, including *hif1aa*, *hif1ab*, *hif1al* and *hif1al2*, but *hif1al2* was the only one up-regulated by hypoxia in this study (Additional file 2). However, *hif1aa*, *hif1ab* and *hif1al* were slightly but significantly induced by cold in our previous study (data not shown). It is interesting that all HIF1α-suppressing genes, including *egln* genes and *hif1an* were induced by hypoxia (Additional file 2) but none of them was up-regulated by cold stress. It is possible that the stress response of hypoxia under 18°C is weaker than that of 5% oxygen and therefore the negative regulatory mechanism is not activated.

The protective effects of hypoxia acclimation against cold stress in zebrafish larvae prompts us to explore the mechanisms activated under both hypoxia and cold. Genes co-induced by hypoxia and cold are mainly involved in oxidation-reduction processes, hemoglobin biosynthetic process and oxygen transport. Cold-induced oxidative stress was widely found in insects [65], fishes [50] and mammals like rat [66], suggesting that establishment of defense systems against oxidative damage would be a common task for cold acclimation in animals. Among the genes co-induced by hypoxia and cold, *SQRDL* (sulfide quinone reductase-like) functions to catalyze the conversion of sulfide to persulfides, thereby decreasing toxic concentrations of sulfide accumulated upon hypoxia/ischemia [67]. HEPHL1 (Hephaestin-like 1) is an analog of ceruloplasmin that functions as a multicopper ferroxidase to convert Fe2^+^ to less toxic Fe3^+^ and aids in counteracting the deleterious effects of iron/ROS-mediated oxidative damage [68]. Cyp46a1 (cytochrome P450, family 46, subfamily A, polypeptide 1) is the cholesterol 24-hydroxylase which catalyzes the oxidation of cholesterol into 24S-hydroxycholesterol to facilitate the efflux of cholesterol across the blood–brain barrier [69]. Since the content of cholesterol is negatively related to the fluidity of cell membrane [70], elimination of cholesterol from cells may be necessary for the increment of membrane fluidity at low temperature. Myoglobin is important for intracellular oxygen transportation upon oxygen scarcity and was reported to be up-regulated by hypoxia in various carp tissues [71,72]. Up-regulation of *mb* by cold stress further suggests that improving oxygen supply is an important aspect of cold acclimation. Furthermore, genes involved in oxygen transport, iron homeostasis and hemoglobin biosynthetic process such as *hbae3*, *tfr1a*, *tfr1b*, *urod* (uroporphyrinogen decarboxylase) and *steap3* (STEAP family member 3, metalloreductase) were up-regulated upon both hypoxia and cold. Nearly all these co-regulated genes are involved in improving cellular oxygen availability and oxidation-reduction process. Furthermore, the results of lipid peroxidation assays demonstrated that both cold and hypoxia pre-acclimation alleviate oxidation damage caused by lethal cold stress, suggesting the association of these genes with cold acclimation. However, the increase in the abundance of transcripts under cold stress is not necessarily resulted from activated transcription since the turnover of RNA can be affected by temperature. The post-transcriptional regulation mechanisms reducing mRNA decay may contribute to the up-regulation of certain genes as well.

In addition to co-induced genes, we identified and confirmed the alternative promoter usage of *hmbsb* under hypoxia and cold. Alternative promoter usage is a versatile mechanism to create diversity and flexibility in the regulation of gene expression besides alternative splicing. Messenger RNA molecules derived from alternative promoters may differ in the level of transcription initiation, stability and translation efficiency. Alternative promoters can have different tissue specificity, react differently to environmental signals and lead to the generation of protein isoforms differing at the amino terminus [73]. Subsequently, a different amino terminus can lead to alterations in protein levels, functions, or subcellular distribution [73]. Zebrafish *hmbsb* gene is involved in the tetrapyrrole biosynthetic process and is specifically expressed in blood [74]. Alternative promoter usage of zebrafish *hmbsb* gene leads to peptides differing in the initial ten amino acids. Promoter transition was also found for human *HMBS* (hydroxymethylbilane synthase) gene: the housekeeping promoter is active in all cells and the alternative promoter is present only in erythroid cells [75]. Although the alternative promoter of human *HMBS* gene is associated with tissue specificity, the biological significance and mechanisms underlying the hypoxia/cold-inducibility of the alternative promoter of zebrafish *hmbsb* gene remains to be characterized.

## Conclusions

Hypoxia acclimation increased the survival rate of zebrafish larvae after lethal cold exposure, indicating that hypoxia-inducible signaling pathways play important roles in the establishment of fish cold resistance. Hypoxia acclimation of zebrafish larvae is intimately associated with biological processes including oxygen transport, oxidation-reduction process, hemoglobin biosynthetic process, erythrocyte development and cellular iron ion homeostasis. Genes co-induced by hypoxia and cold are mainly involved in oxidation-reduction process, oxygen transport, hemopoiesis, hemoglobin biosynthetic process and cellular iron ion homeostasis. An alternative promoter of *hmbsb* is specifically activated by hypoxia and cold. The transcriptional events co-regulated by hypoxia and cold represent the molecular basis of hypoxia-induced protection against cold stress.

## Methods

### Zebrafish larvae and hypoxia exposure

The animal protocol for this study was approved by the Institutional Animal Care and Use Committee of Institute of Hydrobiology (Approval ID: Y21304501). Maintenance of adult zebrafish and embryos were performed as previously described [12,34]. Zebrafish larvae were placed in 60 mm dishes (50 larvae per dish) and were exposed to hypoxia or cold stress at 96 hpf. To investigate the development of cross-resistance to hypoxia and cold, zebrafish larvae were first acclimated to 5% O2 at 28°C (pre-hypoxia) or 18°C in air (pre-cold) for 24 h, the controls were maintained in air at 28°C. After acclimation, larvae were exposed to lethal oxygen (2.5% O2, 28°C) for 5 h or lethal cold (10°C, in air) for 24 h (Figure 1A). Fish with no heart beat and no response to touch were considered as dead. No mortality was observed during the acclimation process. Hypoxia acclimation and challenge were performed in a Ruskinn Invivo2 400 Hypoxia Workstation (Baker). Biochemical incubators (HWS-150, Shanghai Jinghong) were used for temperature control.

### Sequencing library construction and high-throughput sequencing

Total RNA was extracted from hypoxia pre-acclimated and control larvae at 120 hpf using TRIZOL reagent from Invitrogen. All larvae in the same plate were combined and treated as a sample for RNA extraction. The content of RNA was measured using NanoDrop 8000 from Thermo Scientific and the quality of RNA samples was assessed by agarose gel electrophoresis. The integrity of RNA samples was confirmed using Agilent 2100 Bioanalyzer and 4 μg of total RNA was used for isolation of mRNA. Sequencing libraries construction and high throughput sequencing were performed together with our previous study about cold-regulated transcriptome [12]. Briefly, purified mRNA samples were fragmented into small pieces and double-stranded cDNA was synthesized using random hexamer primers. The synthesized cDNA was subjected to end-repair, phosphorylation, 3′ adenylation, adapter ligation and PCR amplification. Finally, sequencing libraries of 250 to 350 bp were constructed. Three independent biological replicates for hypoxia-treated samples were used for library construction. High-throughput sequencing was performed by the Analytical & Testing Center at Institute of Hydrobiology, Chinese Academy of Sciences (http://www.ihb.ac.cn/fxcszx/). Multiplexed libraries were sequenced for 36 bp at both ends using an Illumina Genome Analyzer IIx platform according to the standard Illumina protocols. The datasets have been deposited in NCBI Sequence Read Archive (http://www.ncbi.nlm.nih.gov/Traces/sra, SRA062881). Since the RNA-seq datasets of this study and those of our previous study [12] were generated at the same time, the same controls were used to spare experimental expenditures.

### Bioinformatic analysis

Read filtering and trimming, paired reads extraction, read mapping, transcript assembly, background estimation and differential expression analysis were performed as previously described [12]. Briefly, the preprocessed reads were mapped to the genome sequence of zebrafish (Zv9.69) using TopHat, the assembled transcripts were merged with the reference annotation (Danio_rerio.Zv9.69.gtf, downloaded from Ensembl) using cuffmerge, and differential expression analysis was performed using cuffdiff [53]. The abundance of gene transcripts was expressed as FPKM (Fragments per kilobase of transcript per million fragments mapped) [53]. Genes with a fold change ≥ 1.5 and a q-value ≤ 0.05 were considered to be differentially expressed. Calculation of mapping statistics, sorting and indexing of the read alignment files were performed using SAMtools [76]. The mapping and assembling results were viewed via IGVtools [77]. GO (Gene ontology) enrichment analysis was performed using BiNGO [78], a plugin of Cytoscape [79].

### Quantitative real-time PCR (qPCR)

qPCR analysis was performed according to the MIQE (Minimum information for publication of quantitative real-time PCR experiments) guidelines. Total RNA samples were treated with RNase-free DNase I (Promega) to eliminate contaminated genomic DNA before reverse transcription. First-strand cDNA was synthesized from 4 μg of pretreated total RNA using random hexamer primer with the RevertAid^TM^ First Strand cDNA Synthesis Kit from Fermentas. The PCR primers were designed using Primer Premier 6.0 software. The specificity of candidate primers was checked using Primer-BLAST (http://blast.ncbi.nlm.nih.gov/Blast.cgi) and the secondary structure of amplicons was assessed using the mfold Web Server (http://mfold.rna.albany.edu/?q=mfold). qPCR was performed in a CFX Connect™ Real-Time PCR Detection System (BioRad). The total volume of the reaction system was 20 μL, including 10 μL of 2 × iTaqTM Universal SYBER Green supermix (BioRad), 2 pmol of each primer and 5 μL of 10 × diluted cDNA template. Three independent biological replicates of each treatment were included in the analysis and all reactions were carried out in duplicates. The qPCR amplification program was 95°C for 1 min, followed by 40 cycles of 95°C for 10 sec, 59°C or 60°C for 30 sec (with plate read) and 72°C for 10 sec. The melt curve of PCR product was generated by denaturized at 95°C for 10 sec, heating from 65°C to 95°C with 0.5°C increments and 5 sec dwell time, and a plate read at each temperature. The specificity of the reaction was confirmed by the observation of a single melt peak. The amplification cycle displaying the first significant increase of the fluorescence signal was defined as threshold cycle and used for quantification (Cq).

The standard curve of primers was generated from the Cq values of a series of templates 5 × diluted from the mixture of all samples to be analyzed. The amplification efficiency of primers was calculated from the slope of corresponding standard curve. Information including accession number of genes, amplification efficiency of primers and the length of amplicons were listed in Additional file 7. To identify suitable internal references for qPCR data normalization, commonly utilized reference (*actb1*), previously reported stable reference (*rpl13a*) under hypoxia [80] and genes with smallest expression variations detected using RNA-seq including *ada*, *smarce1*, *erp44*, *ube2e1*, *gnb1b*, *rbx1*, *yipf3* (Additional file 3) were selected as candidate references. A survey of the Gene ontology database indicated that these genes belong to different functional classes. The stability of these genes was measured using geNorm [54] and Normfinder [55], respectively. Moreover, *tpma* and *tnnt3b* were used as internal references for normalization of cold-related qPCR data according to our previous study [34]. qPCR data analysis was performed according to Hellemans et al. [81]. Cq values were converted into relative quantities (RQ) using the gene specific efficiency (E). The geometric mean of RQ values for the selected internal reference genes was calculated and used as the normalization factor (NF). The normalized relative quantity (NRQ) of target genes was calculated by dividing RQ by NF.

### 5′ RACE

5′ RACE was performed to characterize the transcriptional initiation sites using the 5′-Full RACE kit from Takara according to manufacturer’s instruction. This kit uses the decapping method to amplify the full length 5′ sequence of cDNAs. The total RNA samples were first treated with CIAP (Calf intestine Alkaline Phosphatase) to remove the naked phosphorus from incomplete RNA fragments. The CIAP-treated samples were sequentially subjected to decapping and 5′ RNA adaptor ligation using TAP (Tobacco Acid Pyrophosphatase) and T4 RNA Ligase, respectively. Reverse transcription was conducted using M-MLV reverse transcriptase and 9-mer random primer. Finally, 5′ ends of *hmbsb* cDNA were amplified by nest PCR. Primer pairs including 5′ RACE outer primer/hmbsb-5′-R1 and 5′ RACE inner primer/hmbsb-5′-R2 were used for the first and second round PCR, respectively. The sequence of primers was listed in additional file 7. The PCR products were purified using the Biospin Gel Extraction Kit from BioFlux and subcloned into the pMD18-T vector from TaKaRa. The positive clones were selected and subjected to DNA sequencing.

### Lipid peroxidation assay

The content of lipid peroxidation product malondialdehyde (MDA) was measured using the MDA assay kit from Beyotime Biotechnology according the manufacture’s instruction. Larvae at 96 hpf were subjected to mild cold or hypoxia treatment for 24 h. Pre-acclimated larvae were then exposed to 10°C for 12 h. The death rate after such treatment is less than 15%. Larvae in the same plate were combined and homogenized in PBS (pH 7.4) supplemented with 1 mM EDTA and 1 mM PMSF. The homogenates were centrifuged at 4°C, 1600 g for 10 min and the supernatants were used for subsequent analysis. The samples were added with thiobarbituric acid (TBA) solution and the colormetric reaction was performed at 100°C for 15 min. The absorbance was determined using a Spectramax M5 plate reader at 532 nm. The protein concentration of samples was determined by the enhanced BCA protein assay kit from Beyotime Biotechnology and used to normalize the MDA concentration.

### Statistical analysis

Statistical analysis was performed using SPSS 15.0 software for windows. The significant difference in survival rates and MDA concentrations between control and pre-treated samples after lethal hypoxia or cold exposure was analyzed by the Duncan’s multiple range test. The data of gene expression was analyzed by the independent-samples *t*-test. The correlation between the data of RNA-seq and qPCR was analyzed by the Spearman’s rho test.

## Additional files

Additional file 1:
**Exposure of zebrafish larvae to hypoxia inhibited body growth.** **(A)** Photograph of zebrafish larvae after low oxygen (5% O2) exposure. SL: standard length. Red and yellow lines indicate the size of intestine lumen and yolk sac, respectively. **(B)** Body length of zebrafish larvae. Different letters above the bars indicate significant difference (p < 0.05) among treatment groups. Additional file 2:
**Genes regulated by hypoxia.**
Additional file 3:
**Expression of candidate reference genes.**
Additional file 4:
**Identification of most stable reference genes.** **(A)** Average expression stability of candidate reference genes calculated using geNorm. **(B)** Stability value of candidate reference genes analyzed by Normfinder. Additional file 5:
**Results of GO enrichment analysis.**
Additional file 6:
**GO enrichment analysis of hypoxia-inhibited genes.**
Additional file 7:
**Primers used for qPCR and 5′RACE.**
