# Supplementary figures and images for: Transcriptional events co-regulated by hypoxia and cold stresses in Zebrafish larvae
Source: BMC Genomics. 2015 May 15;16(1):385. doi: 10.1186/s12864-015-1560-y (PMC4432979; doi:10.1186/s12864-015-1560-y)

**A**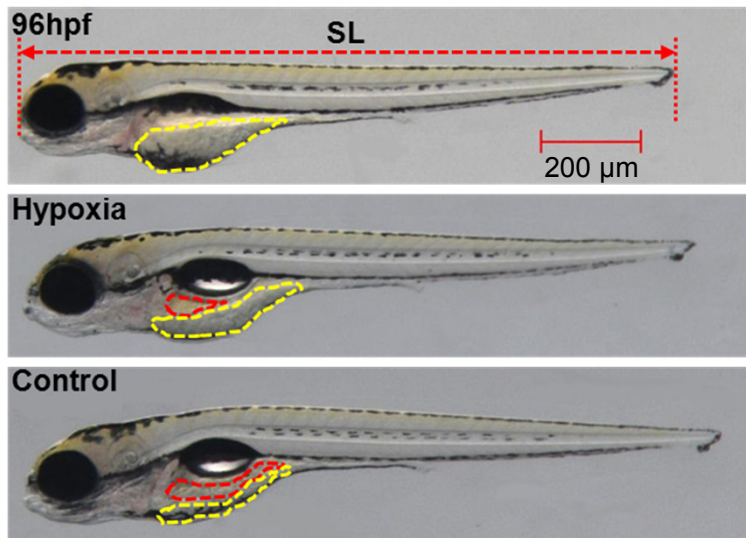**B**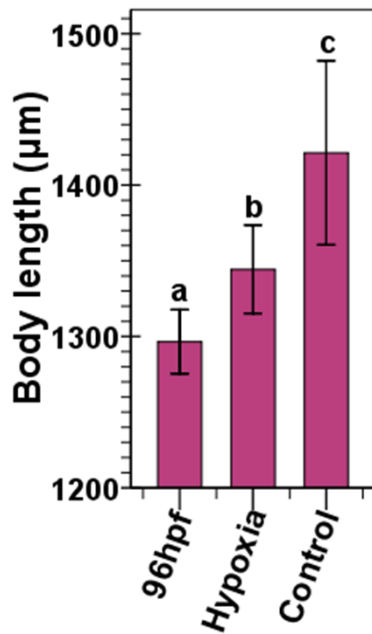

Supplement: Additional file 1: — Exposure of zebrafish larvae to hypoxia inhibited body growth. (A) Photograph of zebrafish larvae after low oxygen (5% O2) exposure. SL: standard length. Red and yellow lines indicate the size of intestine lumen and yolk sac, respectively. (B) Body length of zebrafish larvae. Different letters above the bars indicate significant difference (p < 0.05) among treatment groups. [file 12864_2015_1560_MOESM1_ESM.pdf]

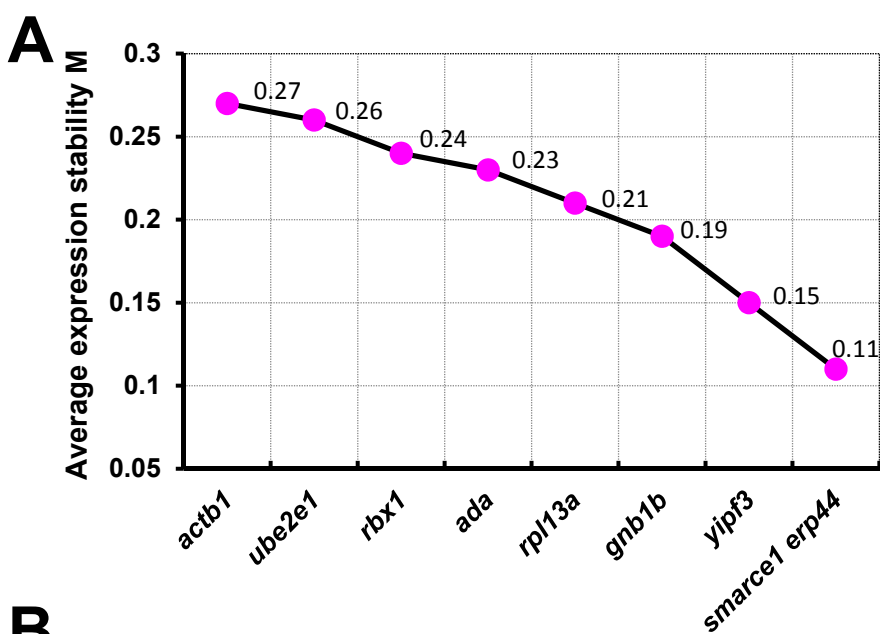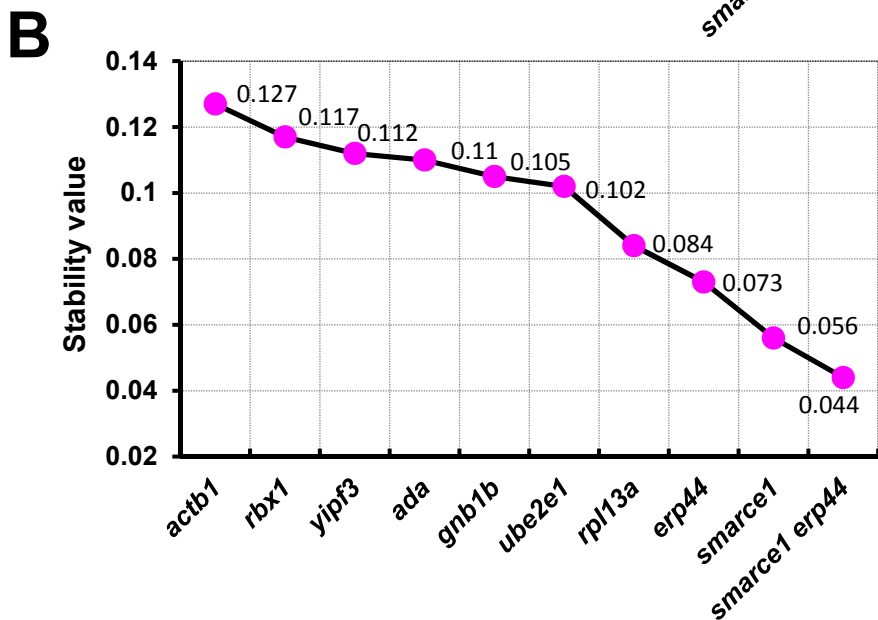

Supplement: Additional file 4: — Identification of most stable reference genes. (A) Average expression stability of candidate reference genes calculated using geNorm. (B) Stability value of candidate reference genes analyzed by Normfinder. [file 12864_2015_1560_MOESM4_ESM.pdf]

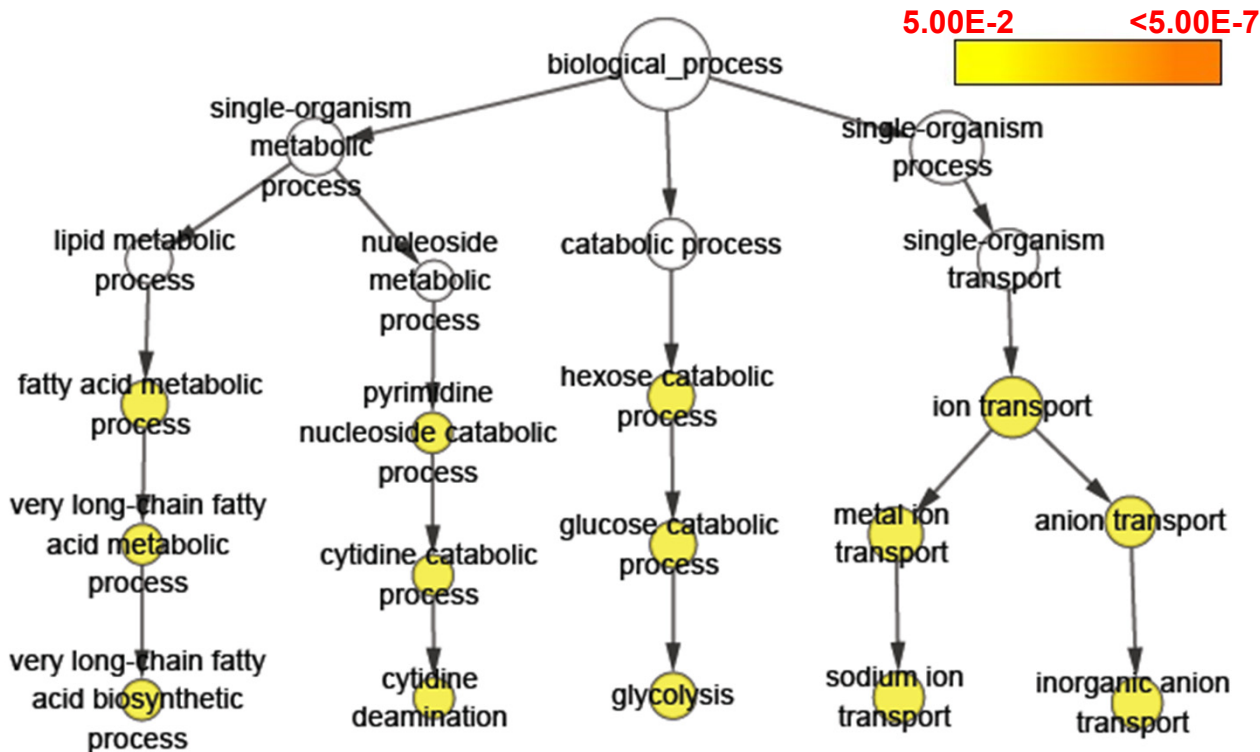

Supplement: Additional file 6: — GO enrichment analysis of hypoxia-inhibited genes. [file 12864_2015_1560_MOESM6_ESM.pdf]
